# Friction stir welding of aluminum alloy 6082-T6 using eccentric shoulder tools to eliminate the need for tool tilting

**DOI:** 10.1038/s41598-025-91065-1

**Published:** 2025-03-14

**Authors:** A. R.S. Essa, A. R.K. Aboud, Mohamed M.Z. Ahmed, A. E El-Nikhaily, Ammar S. Easa, Mohamed I.A. Habba

**Affiliations:** 1 Mechanical Department, Faculty of Technology and Education, Suez University, Suez, 43512 Egypt; 2 Faculty of Engineering, King Salman International University, El-Tor, 45615, Egypt; 3 Department of Mechanical Engineering, College of Engineering at Al Kharj, Prince Sattam Bin Abdulaziz University, Al Kharj, 11942 Saudi Arabia

**Keywords:** Friction stir welding, Eccentric shoulder tool, Tool tilting angle, Mechanical properties, Engineering, Structural materials

## Abstract

The present research investigates the impact of eccentric shoulder tools on the microstructure and mechanical properties of friction stir welded (FSWed) aluminum alloy AA6082-T6. Two tools, one with an eccentric shoulder and one with an aligned shoulder, were employed under identical welding parameters: a rotational speed of 600 rpm, travel speed of 250 mm/min, and tilt angles of 0° and 3°. The four FSWed joints produced were characterized using optical microscopy, tensile testing, and hardness testing. The weld nugget zone (WNZ) microstructure showed significant grain size reduction from 5.24 μm for the base material to 1.63 μm, using the eccentric shoulder tool at 0° tilting angle and 2.78 μm at 3° tilting angle. The aligned shoulder tool resulted in an average grain size of 2.79 μm at 0° tilting angle and 2.23 μm of 3° tilting angle. Thus, the eccentric shoulder tool with a tilt angle of 0° exhibited the smallest average grain size. The mechanical properties obtained are consistent with the microstructure, where the joint produced using the eccentric shoulder at a 0° tilting angle showed the highest tensile strength of 216.5 MPa (89.7% joint efficiency) and 7.71% elongation. In contrast, the aligned shoulder tool resulted in coarser grains and a lower mechanical performance. In addition, this joint exhibited the highest hardness recovery in WNZ. The current study implies that the eccentric shoulder tool can eliminate the need for a tilting angle during FSW, which is required in some applications.

## Introduction

Friction Stir Welding (FSW) is a solid-state joining technique that produces high-quality welds of similar and dissimilar materials^1–4^ It is a new joining technology developed in the 1990s and has since become commonly used in industrial applications^5–7^. FSW involves the use of a rotating tool to generate heat and soften the materials. Heat is generated through direct contact between the rotating tool and material, which is then stirred together to produce a joint^8–11^. FSW is mainly applied to join aluminum alloys and other non-ferrous metals, which can be challenging to weld using fusion welding methods^12–16^. FSW technology has been used in many industrial applications such as aerospace, automotive, and shipbuilding^17,18^. The efficacy of friction stir welded (FSWed) joints depends on a multitude of parameters that must be meticulously established to yield joints that are devoid of defects and possess dependable mechanical properties^19,20^. The key FSW parameters include the rotational speed, welding speed, axial force, tool geometry, and tilting angle^21–24^. The angle of tool inclination is a crucial parameter in the FSW process, necessitating meticulous regulation to attain superior welds because of its impact on the quantity of agitated materials and the heat input throughout the FSW^25,26^. The FSW process involves inclining the rotating tool at a specific angle from the vertical axis, which is known as the tilting angle. The tilting angle is important in determining the quality of the FSW joints^27,28^. Selecting a suitable tilting angle is beneficial because it enables the tool shoulder to make contact with the joint material and promotes the movement of the material around the rotating tool^29^. However, if the tilting angle is excessively large, it can cause the tool pin to rise from the weld root, which can damage the weld^26,29^. Based on previous studies, a tilting angle ranging from 1° to 3° is considered to be appropriate for most materials^25,30^. Long et al.^29^ investigate the impact of a 0 and 2° tilting angle using a 3D thermo-mechanical model and the DEFORM-3D software. Their model considered the tilt angle within the geometrical structure and analyzed two cases with tilt angles of 0 °and 2°. The results showed that the weld had defects in the case of a 0° tilting angle, whereas a 2° tilting angle produced a defect-free weld. Chauhan et al.^31^ study the formation of defects in FSW under three different tilting angles (0, 1 and 2°) using a Coupled Eulerian and Lagrangian (CEL) method and modeling the process with a cylindrical pin in ABAQUS/Explicit. According to their model, a tilting angle of 2° resulted in a weld without defects. By contrast, an increased tilting angle can lead to an increase in the axial force. This is because as the tool tilts, it exerts more force on the metal, requiring a greater force to move it through the material^32^. Also, setting the suitable tilting angle for the materials may require a more complex machine design, with additional axes of motion and more advanced control systems to set the required tilting angles^31,33,34^. This increases the cost of FSW machines. Furthermore, the FSW tool utilized during the FSW process may wear more quickly at higher tilting angles owing to increased heat and friction, particularly because of the FSW of the steel alloys^26,32^. Thus, the present study explores substitutes that curtail the use of the tilting angle in the FSW procedure while mitigating its negative impact on the cost of the FSW machine and tool life. The objective is to achieve this without affecting the quality of the FSW weldments. One of the solutions proposed to achieve the use of a zero tilting angle during the FSW process is the tool geometry. FSW tool geometry is a critical parameter that has an impact on the quality of the FSW joints^35^ and should be designed and selected because of its direct effect on the flow of material and the amount of deformation during the FSW process, which can influence the formation of defects, such as voids and cracks^36,37^. Many studies have studied the effect of tilting angle to solve the problem of defects caused by friction stir welding^26,32,38^. Where the tilting angle increases the strength and torque on the welding joint, which increases the flow of formed materials from front to back and prevents the occurrence of defects^22,25,39^. As the research indicated in their use of the tilting angle, it is in the range of 0° to 4°, which makes the goal of searching for the optimal value of the tilting angle that achieves the best results for mechanical properties and microstructure^40,41^. The lower the tilting angle, the better it is because it reduces the force and torque required for the welding process. It also reduces manufacturing problems on the ground, such as the problems of fixing connections, which require a greater fixation force with the tilting angle owing to the increase in vertical force and torque. On the other hand, with the development of stirred friction welding machines and tools, the simplest designs that help to achieve mobile-stirred friction welding are similar to electric welding and gas welding. The simpler the welding process is, the easier it is to achieve this goal. The objective of this study is to investigate the impact of an eccentric shoulder tool on friction stir welding with and without the application of a tool tilting angle. This is because of the possibility of compensating for the effect of the tilting angle with the eccentric movement of the shoulder, which increases the friction area between the tool and welding joint to achieve the heat generated required to produce an FSWed joint with the highest mechanical properties. Unlike traditional FSW methods, which rely on the tool tilt angle to improve the joint quality, this study demonstrates that an eccentric shoulder tool can eliminate the need for tool tilting, thus simplifying the FSW process without compromising the mechanical properties of the weld.

## Experimental procedure

**Fig. 1:**
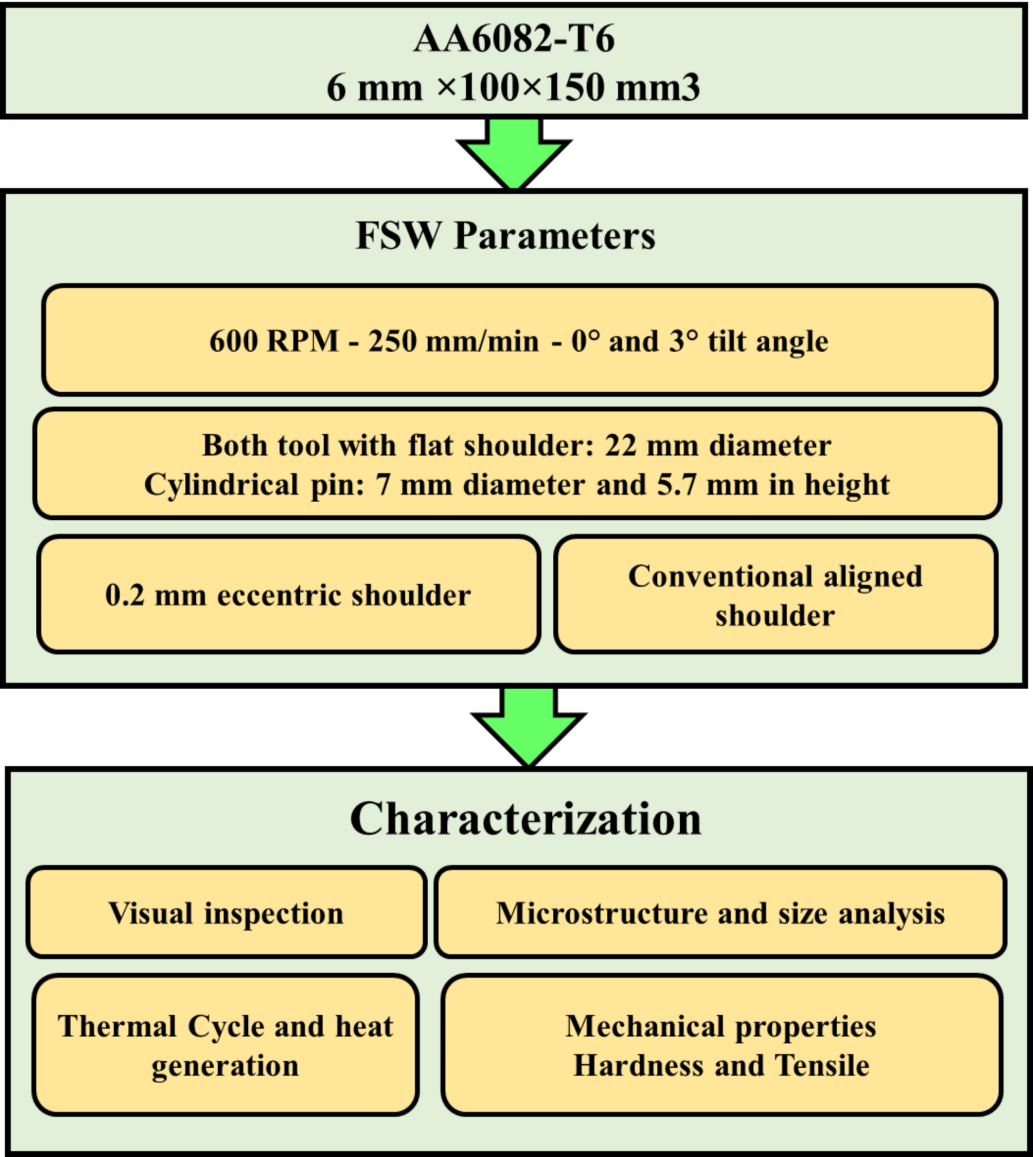
Experimental procedure flowchart of the applied FSW of 6 mm thick AA6082-T6.

**Fig. 2:**
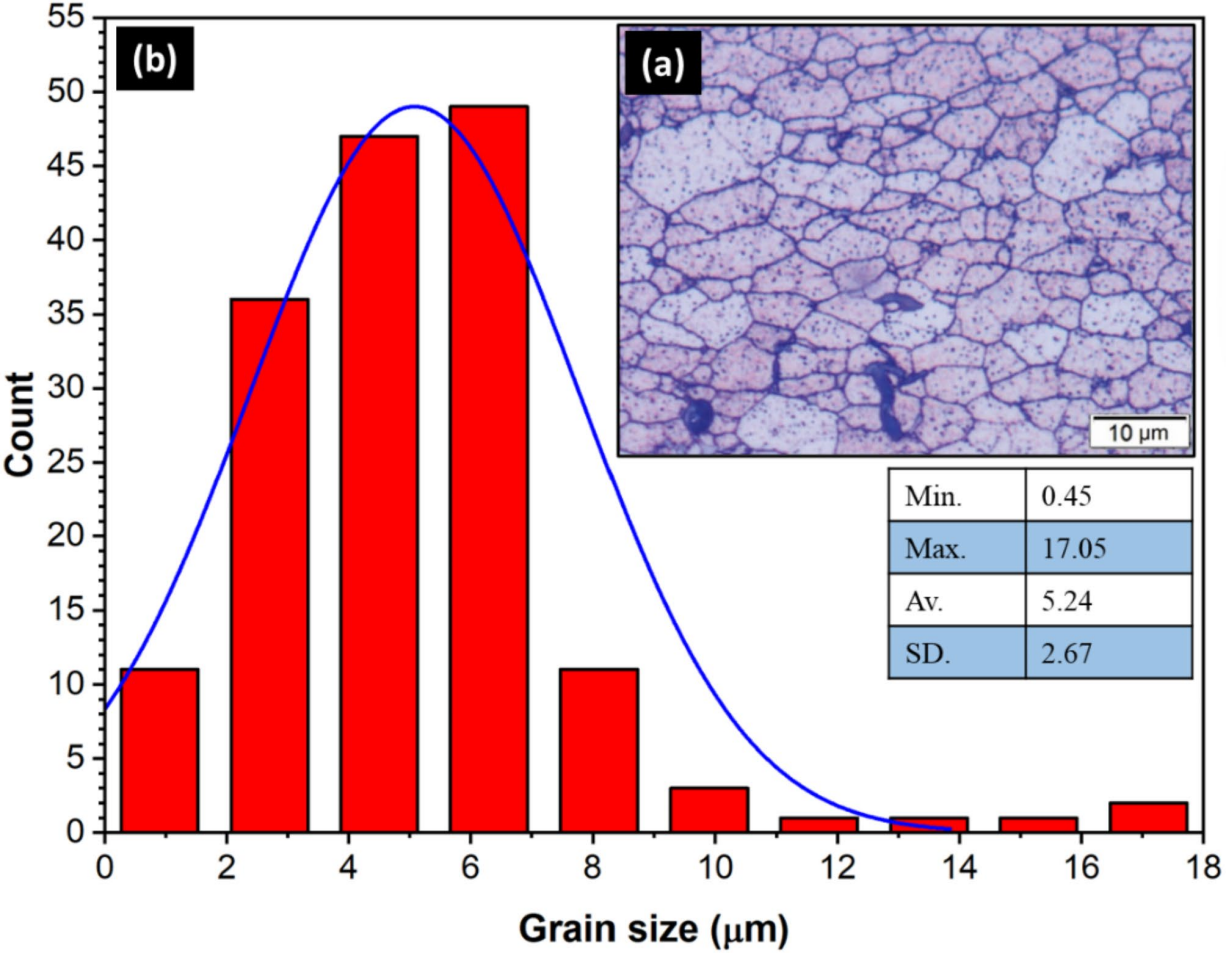
(**a**) Optical microscopy and (**b**) grain size analysis of the AA6082-T6 initial plate.

**Fig. 3:**
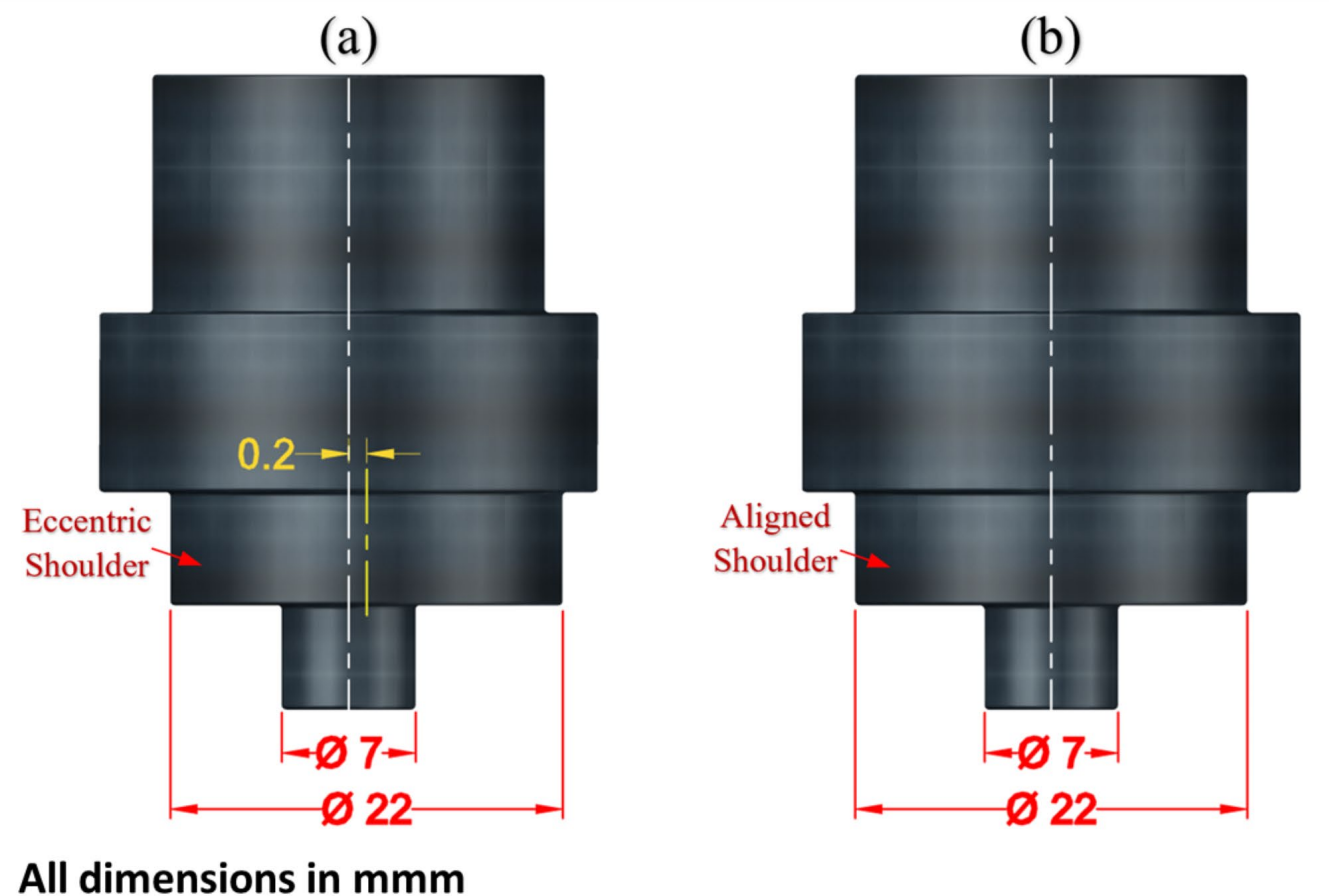
Schematic of the FSW tools: (**a**) eccentric shoulder and (**b**) aligned shoulder.

Figure 1 illustrates the experimental procedure applied for FSW of AA6082-T6. The AA6082-T6 plates used in this study were rolled sheets with a thickness of 6 mm (Composition: Si; 0.70, Fe; 0.05, Cu; 0.10, Mg; 0.60, Mn; 0.40, Cr; 0.25, Zn; 0.20, Ti; 0.10, Al; 97.15, all in wt%) were used for friction stir welding of butt joints. Figure 2a shows the microstructural characteristics and grain size analysis (Fig. 2b), which were evaluated using optical microscopy. The plate measurements were 6 mm ×100 × 150 mm^3^ in size. The FSW tool material employed was a rod made of W302 cold-worked tool steel (5.20% Cr, 0.40% Mn, 0.95% V, 0.39% C, 0.10% Si, 1.40% Mo, and 90.60 wt% Fe), the material was subjected to heat treatment resulting in a hardness of 62 HRC. The tool profile was designed to have a flat shoulder (22 mm diameter) and a 5.7 mm height cylindrical pin (7 mm diameter). Two tools were used for friction stir welding: the first with an eccentric shoulder (0.2 mm as shown in Fig. 3a, and the second with a conventional aligned shoulder, as shown in Fig. 3b.

**Fig. 4:**
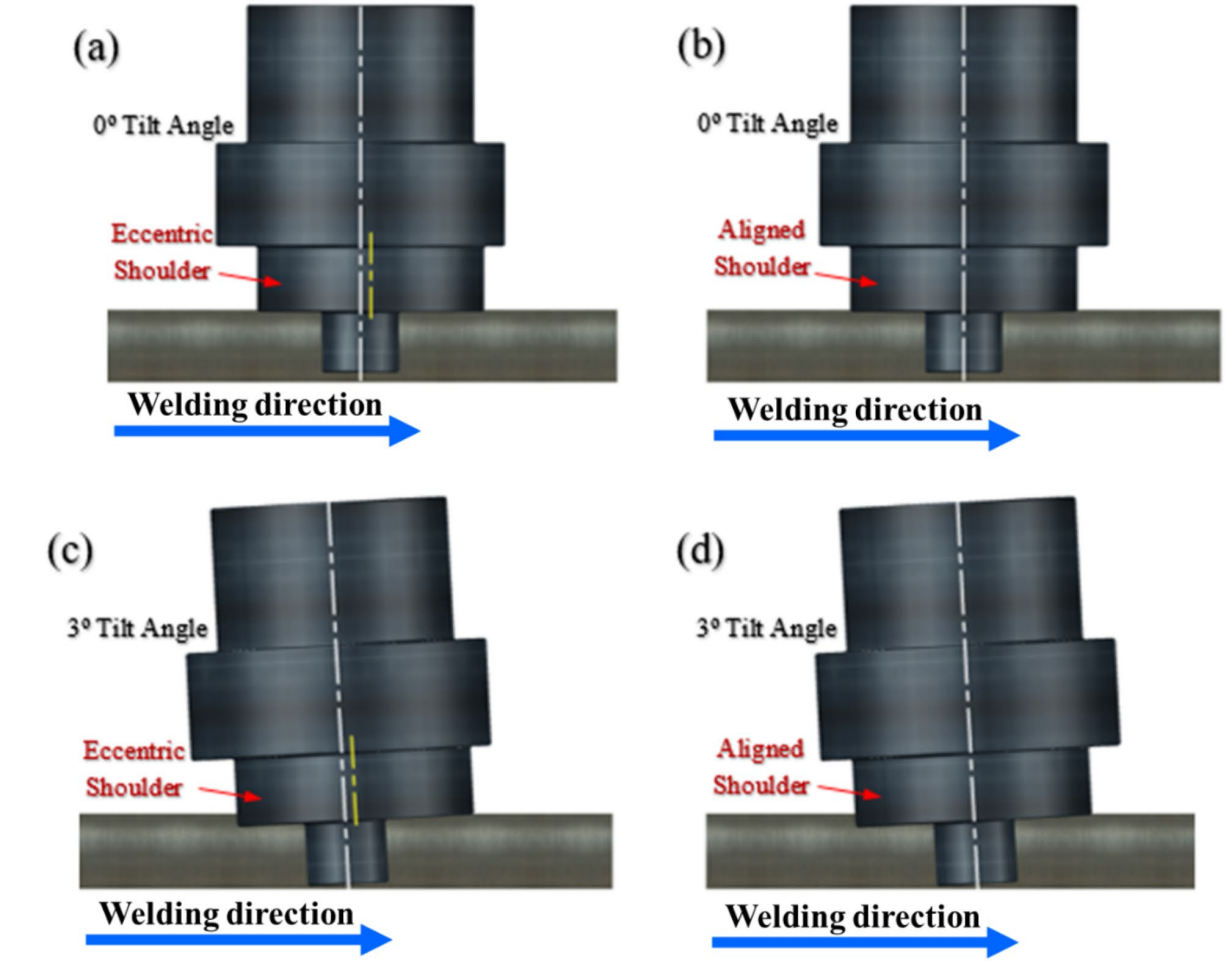
Schematic of FSW processes: (**a**) eccentric shoulder, 0 °tilt angle, (**b**) aligned shoulder, 0 °tilt angle, (**c**) eccentric shoulder, 3 °tilt angle, (**d**) aligned shoulder, and 3 °tilt angle.

The study involved the utilization of a vertical milling machine under standard environmental conditions, with consistent parameters (rotational tool speed: 600 RPM, traverse speed: 250 mm/min, and tilting of the tool by 0° and 3°) for both eccentric and aligned shoulder tools, as shown in Fig. 4a-d. The FSW tool was rotated in a clockwise direction for all welding experiments, and the welding direction was parallel to the rolling direction of the joint plates. The tool penetration depth was set to 5.8 mm to ensure that the shoulder made sufficient contact with the workpiece surface while avoiding excessive plunging. A 3° tilt condition for the eccentric shoulder tool was included to provide a comprehensive comparison across different configurations. This allows for a direct evaluation of the effectiveness of the eccentric shoulder, both with and without tilt. Special attention was paid to comparing the results of the 0° tilt eccentric shoulder configuration with other conditions, particularly the 3° tilt aligned shoulder, which represents a common FSW setup^26,42^. This comparison is crucial for assessing whether the eccentric shoulder can eliminate the need for a tilt angle while maintaining or improving weld quality.

The temperature cycle during the FSW of AA6082-T6 was recorded using an infrared thermometer (Quicktemp 860-T3 model manufactured by Testo Company, Germany. Optical microscopy (OM) was employed to reveal the microstructural characteristics of the FSW joints. Prior to the analysis, the samples underwent a series of preparatory steps, including grinding, mechanical polishing using varying grades of emery papers, and chemical etching with a Keller reagent (190 ml H_2_O, 5 ml HNO_3_, 3 ml HCL, 2 ml HF), and then immersed in the Weck solution (100 mL H_2_O + 4 g KMnO4 + 1 g NaOH) for 7 s to study the microstructure of the FSW joints. Vickers hardness and tensile tests were conducted to investigate the mechanical properties of the specimens. A hardness examination was performed on cross-sectional samples oriented perpendicular to the welding direction and located along the centerline of the cross-section. The examination was conducted using a 1 Kg load. The tensile testing procedure is conducted utilizing an “Instron” tensile testing apparatus with a capacity of 250 kN. The device can operate within the range of 0.0005–1016 mm/min, covering a stroke distance of 1430 mm. The ASTM E8/E8M-16 standard mandates the preparation of flat tensile specimens cut perpendicular to the welding direction. The velocity of the machine head was 0.1 millimeters per second.

## Results and discussion

### Visual inspection

**Fig. 5:**
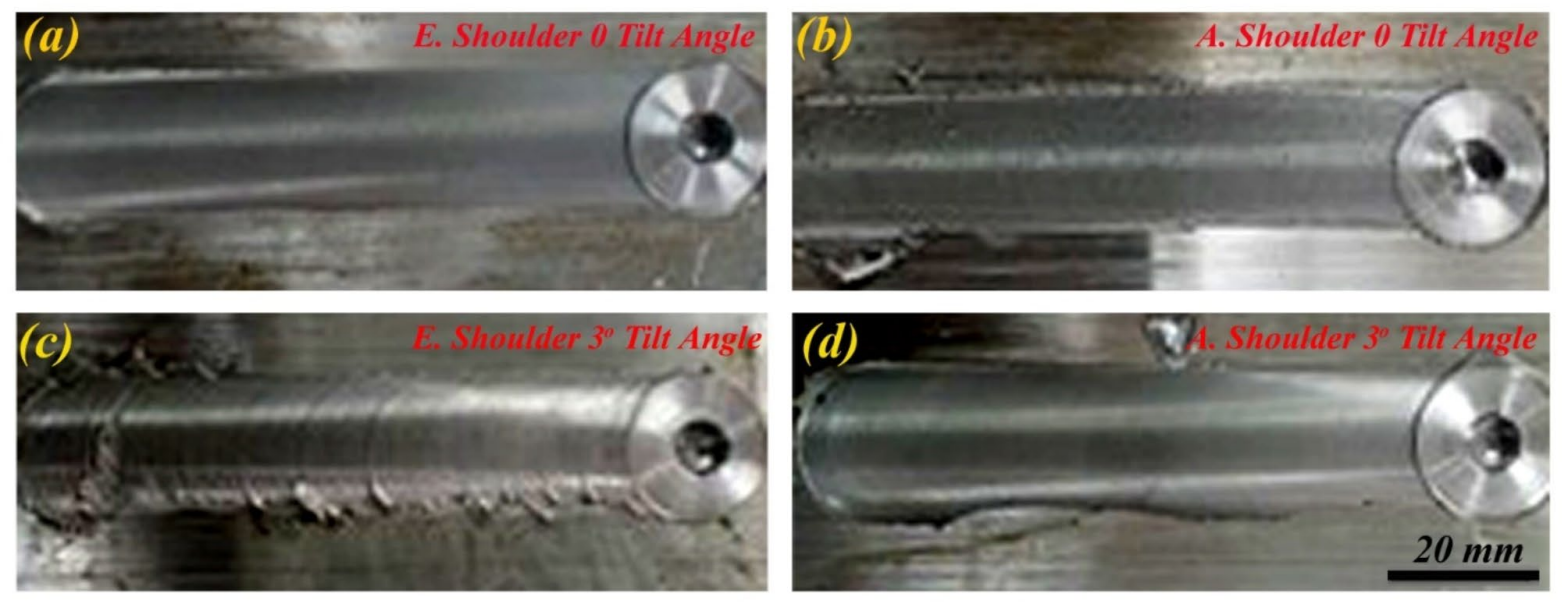
Macrographs of the top surface of the FSWed joints: (**a**) Eccentric shoulder, 0 tilt angle, (**b**) Aligned shoulder, 0 tilt angle, (**c**) Eccentric shoulder, 3^o^ tilt angle, (d) Aligned shoulder, 3^o^ tilt angle.

Photographic images of the FSW joints (top view) are shown in Fig. 5 to detect surface defects of the FSWed joints. FSW surface defects, such as flashes, rough surface textures (ripples), surface grooves, and lack of filling, are common in the FSW process of different materials. One of the critical parameters affecting surface defects is the FSW tool design, which includes shoulder and pin geometry^43,44^. In addition, surface defects at the upper surface of FSWed joints usually start at a low axial force during the FSW process^45^. It can be observed that there were no surface defects, such as surface grooves, and there was a lack of filling of the welded joints using both designed tools, as shown in Fig. 5. However, a flash defect with stretch marks was observed for the joint welded by an eccentric shoulder with a 3^o^ tilting angle, as shown in Fig. 4c, because of the softened material at the contact area between the eccentric shoulder with a tilting angle ejected and extruded on the sides of the welding line to form the flash defect. At the same time, the other welded joints (Fig. 5a, b,d) were formed without flash defects, which indicates that the selection of an appropriate combination between the tool design and tilting angle assisted in filling up the defects by extruding the softened materials downward instead of ejecting them up to the sides of the weld surface^46^. Moreover, the surfaces had the same features as typical FSWed/processed surfaces, such as ripples and keyholes. For ripples, the rotating tool shoulder moves along the joint surface, leaving behind markings called ripples that affect the rough texture of the surface. Figure 5 shows the delicate ripples that indicate a high number of rotations per interval. For ripples, the distance between the two ripples depends on the combination of the travel and rotation speeds applied^47,48^. Li et al.^48^ observed that the spacing between ripples is directly related to the ratio of welding speed to rotational speed. This observation is consistent with the observations of the current study. The ripple patterns visible on the weld surfaces (Fig. 5) were a result of the tool rotation and translation during the welding process. The fine ripples observed indicate a high number of rotations per unit distance traveled, which aligns with the welding parameters used (rotational speed of 600 rpm and travel speed of 250 mm/min).

On the other hand, the formation of the keyhole is one of the significant features of the FSW process. The keyhole was a depression in the welding zone formed by the rotating pin after exiting the welded plates. This feature is critical because it provides a visual indicator of the quality of FSWed joints. If the keyhole is correctly formed, it indicates a strong and effective weld. If a keyhole is not correctly formed, it can reveal weaknesses or defects in the welded joint. Thus, the keyhole is an essential feature of the FSW process that assists in ensuring the quality of the produced joints. Figure 5 shows the properly formed keyholes for all applied FSW parameters at a rotation speed of 600 rpm and travel speed of 250 mm/min using a 3° tilting angle for both the designed tools of the aligned and eccentric shoulders. Table 1 lists the visual inspection reports of the FSWed joints using aligned and eccentric shoulders with and without tilting angles. All welded joints were visually accepted on both sides of the joints.

**Table 1:** Visual inspection report of the FSWed joints.

Item	Sample No.	Welding Process	Description (mm)	Evaluation	Remarks
1	E0	FSW using cylindrical Cy pin	L 200 × 150 × 5	Accepted both sides	-
2	A0		L 200 × 150 × 5	Accepted both sides	• A little flash needs to be removed.
3	E3		L 200 × 150 × 5	Accepted both sides	• Much flash needs to be removed.
4	A3		L 200 × 150 × 5	Accepted both sides	• A little flash needs to be removed.

### Heat input & measured temperature

**Fig. 6:**
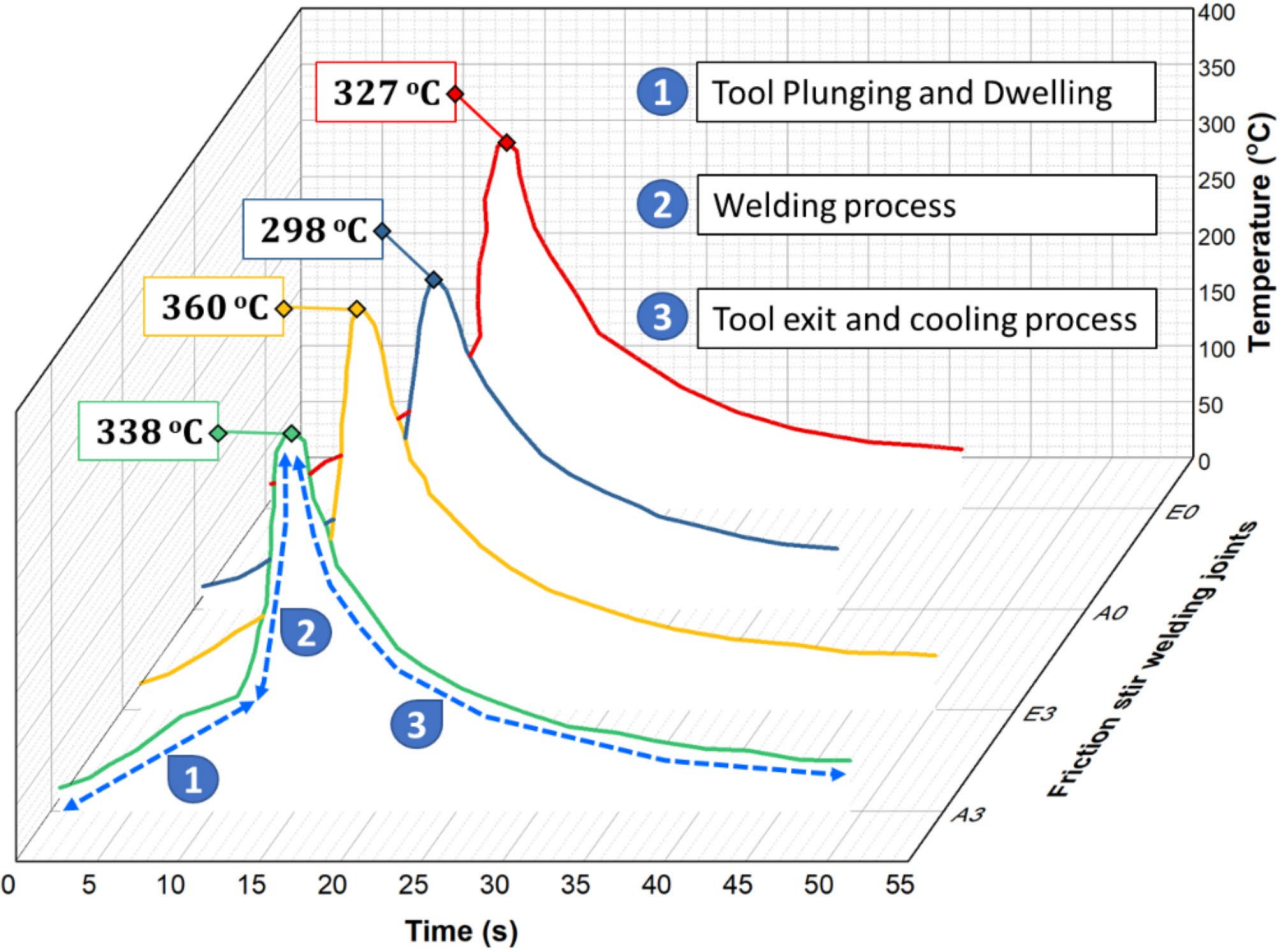
Thermal cycles of the FSW joints using aligned and eccentric shoulders with and without a tilt angle of 3^o^.

The thermal cycle history during the FSW process is essential for detecting the quality of the welded joint by controlling the FSW process parameters, such as the rotation speed, travel speed, and tool geometry, including the pin profile, shoulder features, and tilting angle. Figure 6 plots the thermal FSW cycles on the welding path center recorded using the infrared device for welded joints E0, A0, E3, and A3. The thermal cycles of the welded joints had the same profile and could be divided into three regions: (1) plunging and dwelling, (2) welding, and (3) retracting and cooling, as shown in Fig. 6. In the first region, the rotating tool is inserted into the joint plates to penetrate the plates, generating heat and plastic deformation owing to frictional contact between the tool and the joint plates. During the dwell stage, the rotating tool remained motionless to allow the generated heat to disperse evenly across the weld zone. In the second region, in the welding stage, the rotating tool moved along the joint plates in the welding area, and the heat generation process and stirring action continued. The heat generated in the weld zone reaches its peak point, forming a softened region known as the “thermomechanical affected zone” (TMAZ) on the advanced and retreating sides. Finally, the rotating tool was withdrawn from the welded plates in the tool-exit stage, and the welded joints were cooled to room temperature. It can be noticed that the highest peak temperature of 360 °C recorded during the FSW process of AA6082-T6 is recorded for the E3 joint welded using the eccentric shoulder with a 3° tilt angle. The lowest peak temperature of 298 °C was measured for the A0 joint produced using an aligned shoulder with a tilting angle of 0°. To better understand the effect of using an eccentric shoulder tool on the properties of FSWed joints, the heat generated from different tools is given by the following equation:^49^.

$$\:{Q}_{Energy/Length}=\frac{2}{3v}\pi\:\bullet\:\omega\:\bullet\:{\tau\:}_{contact}\left[{R}_{S}^{3}+3{H}_{p}{R}_{p}^{2}\right]$$

**Fig. 7:**
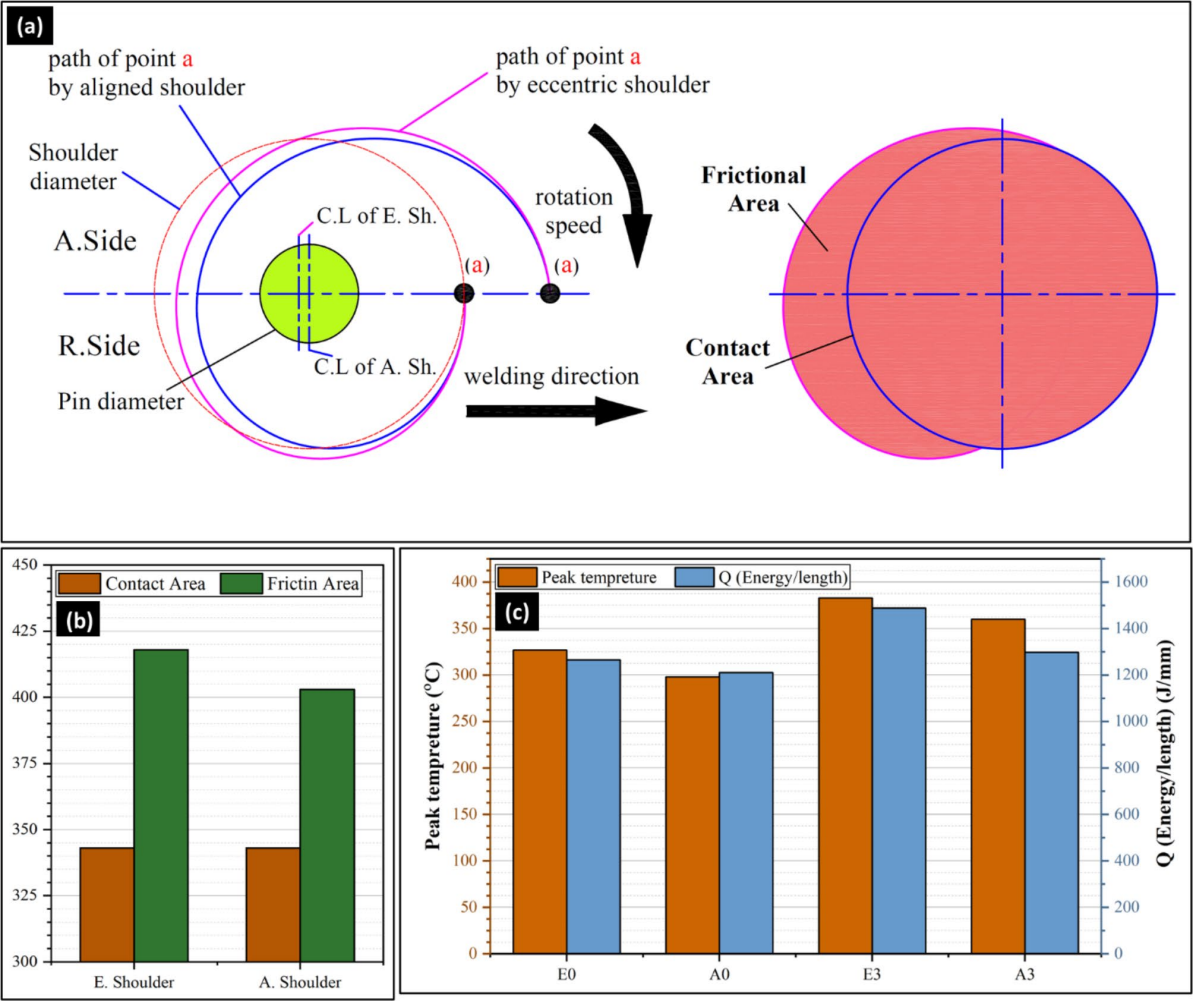
(**a**) Sketch of eccentric shoulder paths of point “a,” (**b**) contact and friction areas of eccentric and aligned shoulders, and (**c**) M-measured peak temperature and calculated heat generation (Q_Energy/length_) for FSW joints.

Figure 7a illustrates the eccentric shoulder paths of point “a” produced by tools with shoulder eccentricities of 0, and 0.2 mm. It can be noted that the tool with a 0.2 mm eccentric shoulder provides a larger friction area between the tool and the materials than an aligned shoulder (Fig. 7b). This is because the eccentric part of the tool creates a dynamic orbit during rotation, which increases the effective area of interaction between the tool and workpiece^19^. The larger surface area of the eccentric shoulder can concentrate more work in the plasticized region, dragging more material and potentially leading to a wider stir zone compared with the aligned shoulders. Figure 7c shows the effect of the eccentric shoulder on the peak temperature and the calculated heat generation. First, it can be remarked that the eccentric shoulder generally produces higher heat generation and peak temperatures than the aligned shoulder (Fig. 7c). This is primarily due to the increased friction area between the tool shoulder and workpiece material, and the dynamic orbit created during tool rotation and traveling. The larger effective interaction area of the eccentric shoulder led to more material flow, particularly under the tool shoulder, and caused periodic changes in the flow velocity at the WNZ. These factors contribute to the higher heat generation and temperature, especially under the shoulder. Second, the heat generation and peak temperature increased with increasing tilting angle from 0 °to 3° for both eccentric and aligned shoulders (Fig. 7c). The tilting angle affects the heat distribution across the weld, and more heat is generated at the trailing edge of the shoulder when the tilting is applied. The tilted tool created increased friction and material deformation, particularly on the advancing side, resulting in a higher peak temperature^50^. Zhai et al.^51^ found that a tilted tool generally leads to higher heat generation than a non-tilted tool (0°), with more heat generated at the trailing edge of the shoulder. Zhang et al.^52^ observed higher temperatures on the advancing side with tilted tools, which was attributed to incomplete contact at the shoulder/workpiece interface. Meyghani and Awang^42^ investigated the influence of the tilting angle on the thermomechanical behavior in the FSW of aluminum 6061-T6, observing increases in the welding temperature with tilting angles compared to the tool and without tilting angle. It is important to note that the peak temperature using an eccentric shoulder tool for a 0° tilting angle exceeds that of the aligned shoulder tool by approximately 10% for the same welding parameters and tilting angle. It is clear that both the energy per unit length and peak temperature increase with an increase in the tilting angle to 3° using the two tools. It is important to note that the peak temperature using the eccentric shoulder tool and 0° tilting angle exceeded approximately 10% over the aligned shoulder tool for the same welding parameters and tilting angle. This implies that the heat input available for sound joint formation is slightly higher for the eccentric shoulder tool than for the aligned shoulder tool. This could be an important concept for producing high-strength FSW joints when the energy per unit length is increased.

### Microstructure

**Fig. 8:**
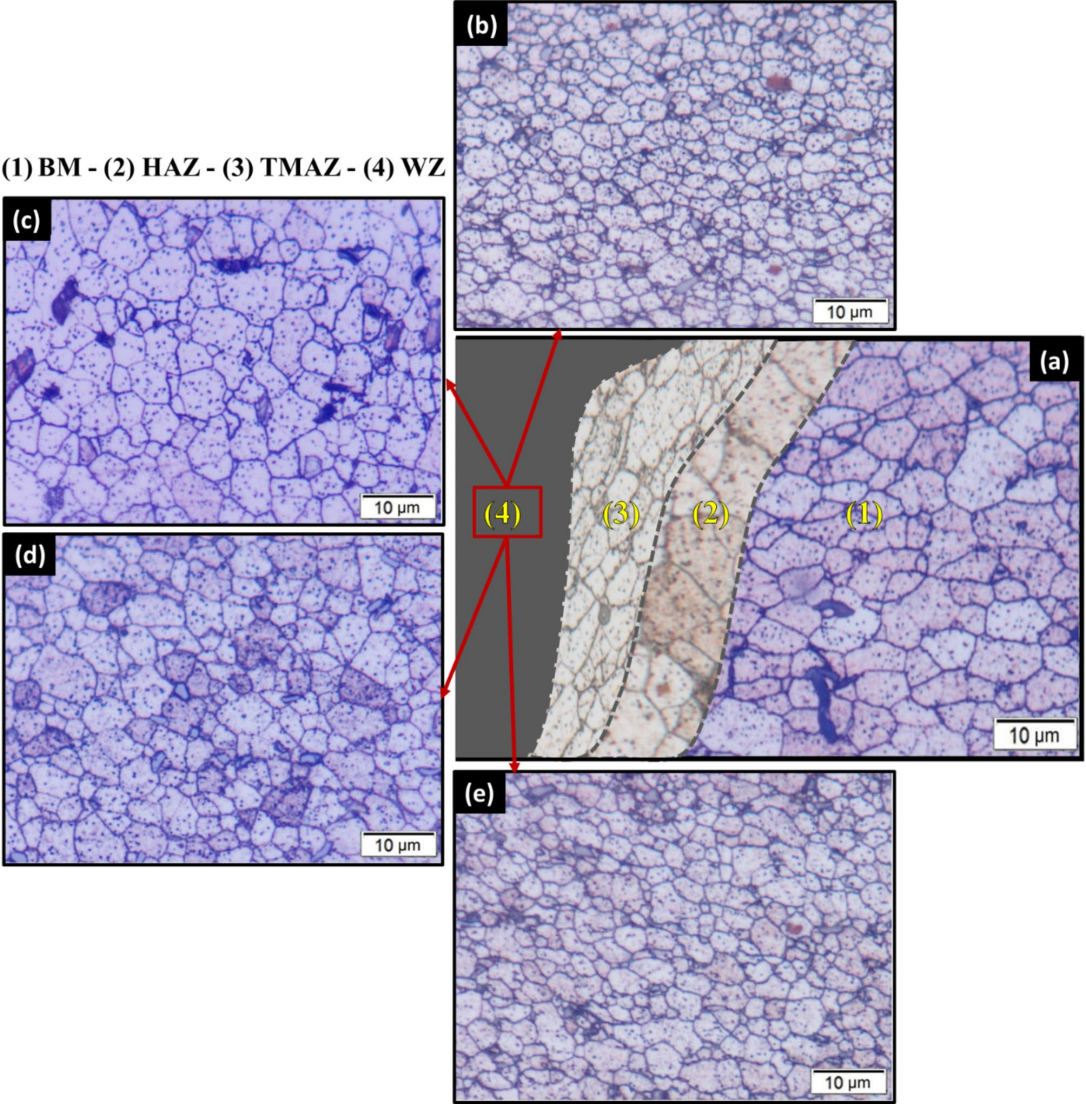
Microstructures in AA6082 FSWed joints of (**a**) BM, HAZ, and TMAZ zones. and microstructure of nugget zone for (**b**) E0, (**c**) A0, (**d**) E3, and (**e**) A3 joints.

**Fig. 9:**
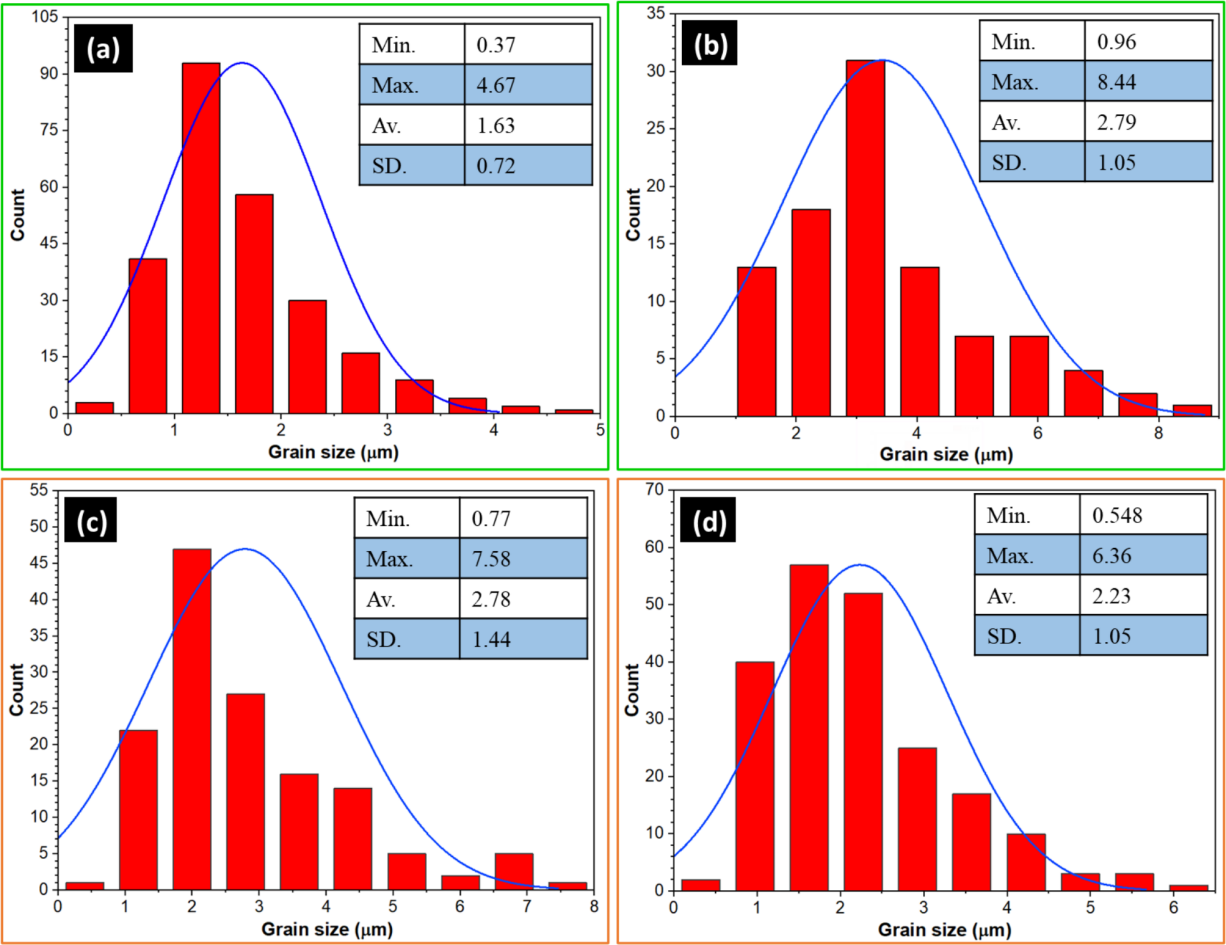
The grain size analysis of the FSWed joints of (**a**) E0, (**b**) A0, (**c**) E3, and (**d**) A3.

Figure 8 depicts the nugget zone microstructure of the FSWed joints of (a) E0, (b) A0, (c) E3, and (d) A3. Figure 9 shows the grain-size distribution histograms of the FSWed joints. In the FSW-welded joints of AA6082, the microstructures in the initial plate, HAZ, and TMAZ exhibited distinct characteristics owing to the welding process. The AA6082-T6 initial plate retained its original microstructure, characterized by larger grains, because it was not subjected to welding heat or deformation, as shown in Fig. 8. In contrast, the HAZ experienced thermal exposure without mechanical deformation, leading to grain growth and coarsening compared with the initial plate (Fig. 8**)**. The TMAZ, located between the HAZ and weld nugget zone, undergoes thermal and deformation effects, resulting in a deformed microstructure with elongated grains. Dynamic recrystallization in the TMAZ was incomplete, leading to a mixture of recrystallized and deformed grains, as depicted in Fig. 8. The nugget zone of all welded joints (Fig. 8b-e) was indicated by equiaxed fine grains compared to the initial material (Fig. 2) for all FSWed joints owing to dynamic recrystallization. Dynamic recrystallization occurred during the FSW process, including the formation of new grains in the weld nugget zone. During FSW, the heat generated by the frictional contact between the rotating tool and joint plates softens the materials in the stir zone, allowing them to flow and consolidate under the pressure of the rotating tool^53,54^. This plastic deformation (stirring action) formed new grains owing to the dynamic recrystallization process. This process plays an essential role in governing the microstructure of the weld nugget zone (WNZ) and can significantly affect the mechanical properties of the welded joints. Several studies have investigated the effects of dynamic recrystallization on the microstructure of FSW joints. They reported that the amount of dynamic recrystallization could be governed by welding parameters such as rotation speed, travel speed, tilting angle, and tool geometry^55,56^. Prangnell and Heason^56^ observed that dynamic recrystallization in aluminum alloys during FSW occurs through a continuous mechanism, with new grains formed by the progressive rotation of subgrains. They found that the welding parameters significantly affected the extent of dynamic recrystallization and grain size. Su et al.^55^ studied the effect of tool rotation speed on dynamic recrystallization in FSW of AA7050 aluminum alloy, noting that higher rotation speeds led to more extensive dynamic recrystallization and finer grain sizes in the nugget zone. They also observed that the size and distribution of recrystallized grains developed during the FSW process could influence the mechanical properties of FSWed joints. In the current study, the results of the developed microstructure show that the heat generated during the FSW process affected by the tilting angle (0 °or 3°) and shoulder geometry (eccentric or aligned) significantly influences the grain size of the WNZ during the FSW process. The average recrystallized grain size was around 1.63 ± 0.7 μm, 2.79 ± 0.4 μm, 2.78 ± 0.4 μm, and 2.23 ± 0.5 μm for E0, A0, E3, and A3 joints, respectively (Fig. 9). In addition, the highest reduction in the average grain size was observed in the joints of E0 (approximately 69%) compared to the AA6082-T6 initial plate.

### Mechanical properties

**Fig. 10:**
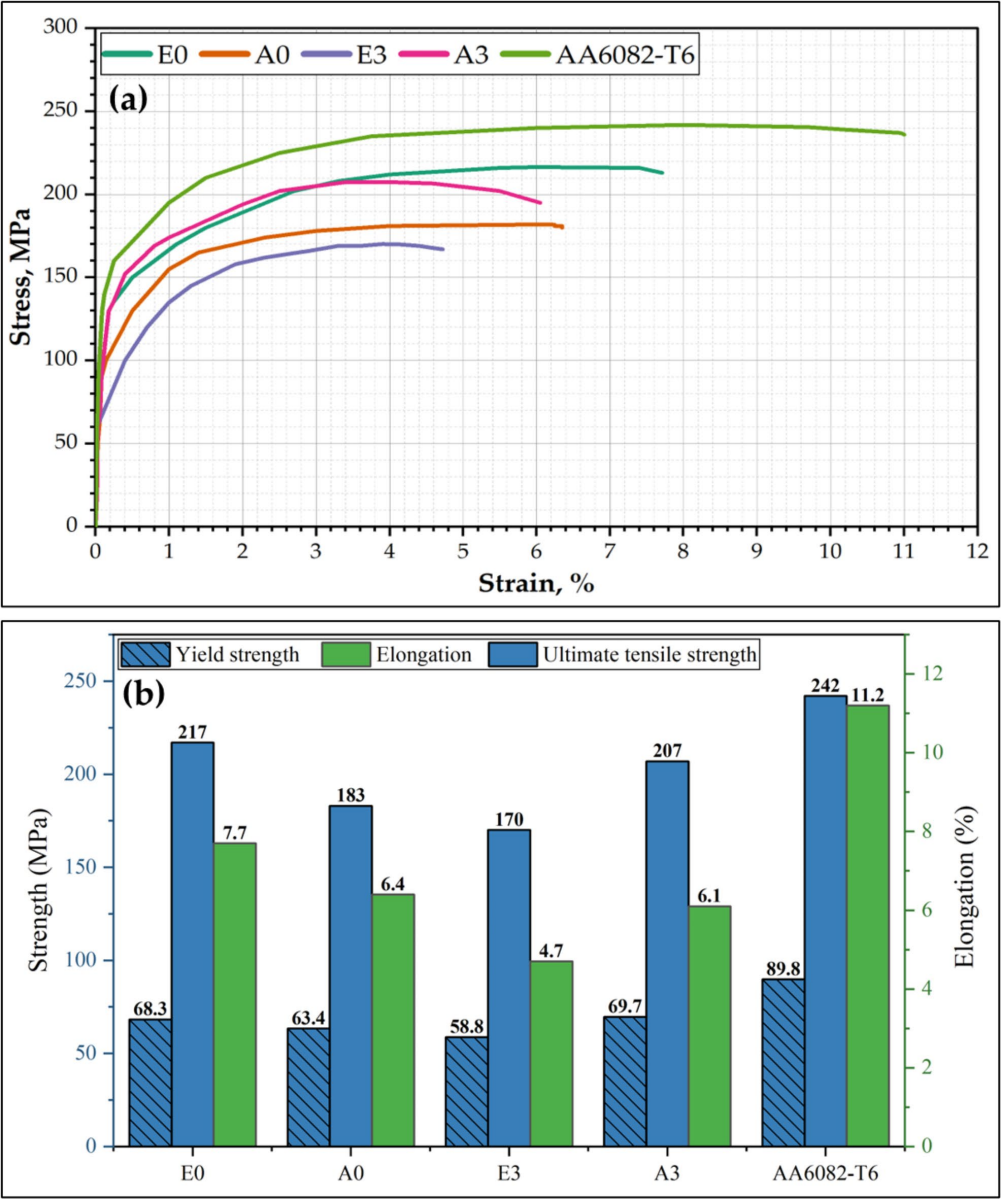
Mechanical properties of FSWed joints: (**a**) stress-strain curves and (**b**) tensile properties of ultimate tensile strength and elongation.

**Fig. 11:**
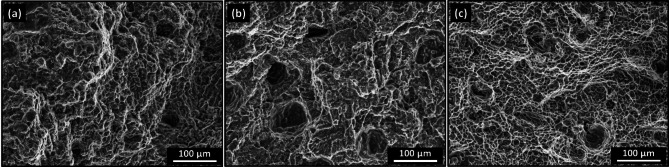
Fracture surfaces of the tensile tested specimens of (**a**) AA6082-T6 initial plate and FSWed joint s of (**b**) E3, and (**c**) E0.

The effect of eccentric and aligned shoulder tools with and without a tilting angle on the tensile properties of the FSWed butt joints was tested using transverse tensile samples containing WNZ at the center. The engineering stress-strain curves of the tensile-tested specimens of the welded joints and Aa6082-T6 initial plate are plotted in Fig. 10a. The results, including the tensile strength, yield strength, and elongation of the tensile tests, are compared in Fig. 10b, which shows the average values of the three tested samples. For the joints welded without a tilting angle, the tensile strength of the welded joint using the eccentric shoulder (E0) was higher than that of the aligned shoulder (A0), as shown in Fig. 10a. The E0 and A0 joints exhibited tensile strength of 216.5 and 183 MPa, respectively, with a joint efficiency of over 90% and 75% of the tensile strength of the initial material (Fig. 10b). On the other hand, for the FSWed joints produced using a tilting angle of 3°, joint E3 exhibited a tensile strength of 170 MPa with 70% efficiency, and the tensile strength increased to 208 MPa with 86% efficiency when the aligned shoulder was applied (Fig. 10b). In addition, the elongation property of the welded joints has the same trend as the tensile strength, which is the highest elongation observed for E0 (highest tensile strength) and the lowest elongation detected for E3 (lowest tensile strength). For joints welded without a tilt angle, the E0 joint exhibited superior mechanical properties compared to the A0 joint. The E0 joint achieved a yield strength of 68.3 MPa, 76% of the 89.8 MPa for the AA6082-T6 initial plate. In contrast, the A0 joint showed a yield strength of 63.4 MPa, corresponding to 70.6% of the base material. Interestingly, for the FSWed joints produced using a tilt angle, A3 joint outperformed E3 joint regarding yield strength. The A3 joint demonstrated the highest yield strength among all welded joints at 69.7 MPa, which is approximately 77.6% of the initial plate, while the E3 joint had the lowest at 58.8 MPa, which is approximately 65.5% of the initial plate. Compared to the welded joints, the E0 joint (eccentric shoulder and 0° tilting angle) achieved a smaller grain size of 1.63 (Figs. 8 and 9). The grain size of the nugget zone significantly affects the mechanical properties of welded joints, including their strength and ductility. In general, smaller grain sizes result in a higher strength and lower ductility. This is because smaller grains provide more hindrances to dislocation motion and improve the tensile strength of the joints but also make it more difficult for the welded material (nugget zone) to deform during the tensile test, which reduces the ductility. Chen et al.^57^ investigated dissimilar AA6061 and AA7075 joined using single-pass and multiple-pass FSW to investigate the effects of a second overlapping pass and its welding direction on the NZ. Compared to single-pass FSW, multiple-pass FSW resulted in finer grains (20% decrease in average grain size) and increased high-angle grain boundaries (2% increase) in the NZ owing to the prolonged dynamic recrystallization. Reversing the welding direction of the second pass enhanced vertical material flow, leading to even finer grains (from 4.2 μm to 2.1 μm) and improved mixing of the dissimilar alloys. The NZ was strengthened after the second pass, with a 3% increase in the yield strength and ultimate tensile strength compared to the single-pass FSW. Multiple-pass FSW with opposite welding directions produced the highest tensile strength, with the yield strength and ultimate tensile strength increased by over 50/30 MPa compared to single-pass FSW. Robert Kosturek et al.^58^ examined the mechanical properties and microstructure of 5-mm-thick AA7075-T65 FSWed joints. They reported that a finer grain size in the WNZ was correlated with improved mechanical properties. The highest ultimate tensile strength of 447.7 MPa (76.7% joint efficiency) was achieved for the joint produced using a tool rotation speed of 400 rpm and 100 mm/min welding velocity, which resulted in a WNZ grain size of 5.2 ± 1.7 μm. This joint also exhibited a higher microhardness than joints with higher heat input parameters. Their research demonstrated that a lower heat input (lower rotation speed and higher welding velocity) resulted in a finer grain size in the WNZ and improved the mechanical properties of the FSWed joints. Moustafa et al.^59^ investigated the microstructural and mechanical properties of dissimilar AA7075 and AA2024 aluminum alloys. They concluded that the FSW process resulted in a fine-grained structure in the stirred zone, with a significant reduction in grain size compared with the base AA7075 and AA2024 aluminum alloys. The average grain size reduced from to 80–180 μm in the initial plates to 12 ± 4 μm in the weld zone. The tensile strength improved with increasing tool rotation speed, and the tensile strength increased by 7.1% when the speed increased from 400 to 560 rpm and by 5.4% when the speed increased from 560 to 700 rpm. Microstructural analysis revealed the formation of fine equiaxed grains in the WNZ, suggesting that the fine-grained structure contributed to these mechanical properties. In this study, compared to the AA6082 initial plate, the E0 joint exhibited the best mechanical performance among the welded samples, with 89.7% UTS and 68.8% elongation, with the finest grain structure having an average grain size of 1.63 μm. In contrast, the E3 joint with a grain size of 2.78 μm showed the lowest mechanical performance, despite having a grain size similar to that of A0. This suggests that factors beyond the grain size, such as the precipitate distribution or residual stresses, may also play a role in determining the final mechanical properties of FSW joints^57,60^. The fracture locations of tensile-tested specimens were analyzed to understand failure mechanisms in friction stir welded joints. For all joints (E0, A0, E3, and A3), fractures consistently occurred in the TMAZ on the advancing side. This can be attributed to the TMAZ experiencing significant thermal exposure during welding without sufficient dynamic recrystallization, resulting in a mixture of deformed and partially recrystallized grains^61,62^. This localized softening reduces its load-bearing capacity. Furthermore, the advancing side undergoes higher material flow and strain rates due to tool rotation and translational motion^63,64^. This leads to greater stress concentrations during tensile loading, making it more prone to failure. In addition, the grain structure in the TMAZ is elongated and less refined (Fig. 8) compared to the WNZ, contributing to reduced hardness. Additionally, precipitate dissolution and coarsening in this region further degrade its mechanical properties. The fracture surfaces of the tensile-tested specimens are depicted in Fig. 11 for the AA6082-T6 initial plate and FSWed joints E3 and E0. The fracture surface of the AA6082-T6 initial plate (Fig. 11a) exhibited a ductile fracture pattern characterized by large and deep dimples (blue area in Fig. 11a). In contrast, E3 joint (Fig. 11b), welded using an eccentric shoulder tool with a 3° tilting angle, shows a mixed fracture mode with both ductile and brittle features. Flat facets alongside shallow dimples (blue and yellow areas in Fig. 11b)suggest that E3 joint experienced plastic deformation and brittle fracture. Meanwhile, the E0 joint (Fig. 11c), created with an eccentric shoulder tool and a 0° tilting angle, predominantly displayed a ductile fracture surface with more uniform, finer, and deeper dimples. This indicates a better plastic deformation and suggests that the welding parameters for E0 resulted in a more homogeneous and ductile weld zone, likely because of the finer grain structure in the WNZ (Figs. 8 and 9). This finer grain structure contributes to the improved ductility and mechanical performance of the E0 joint compared to those of the E3 joint.

**Fig. 12:**
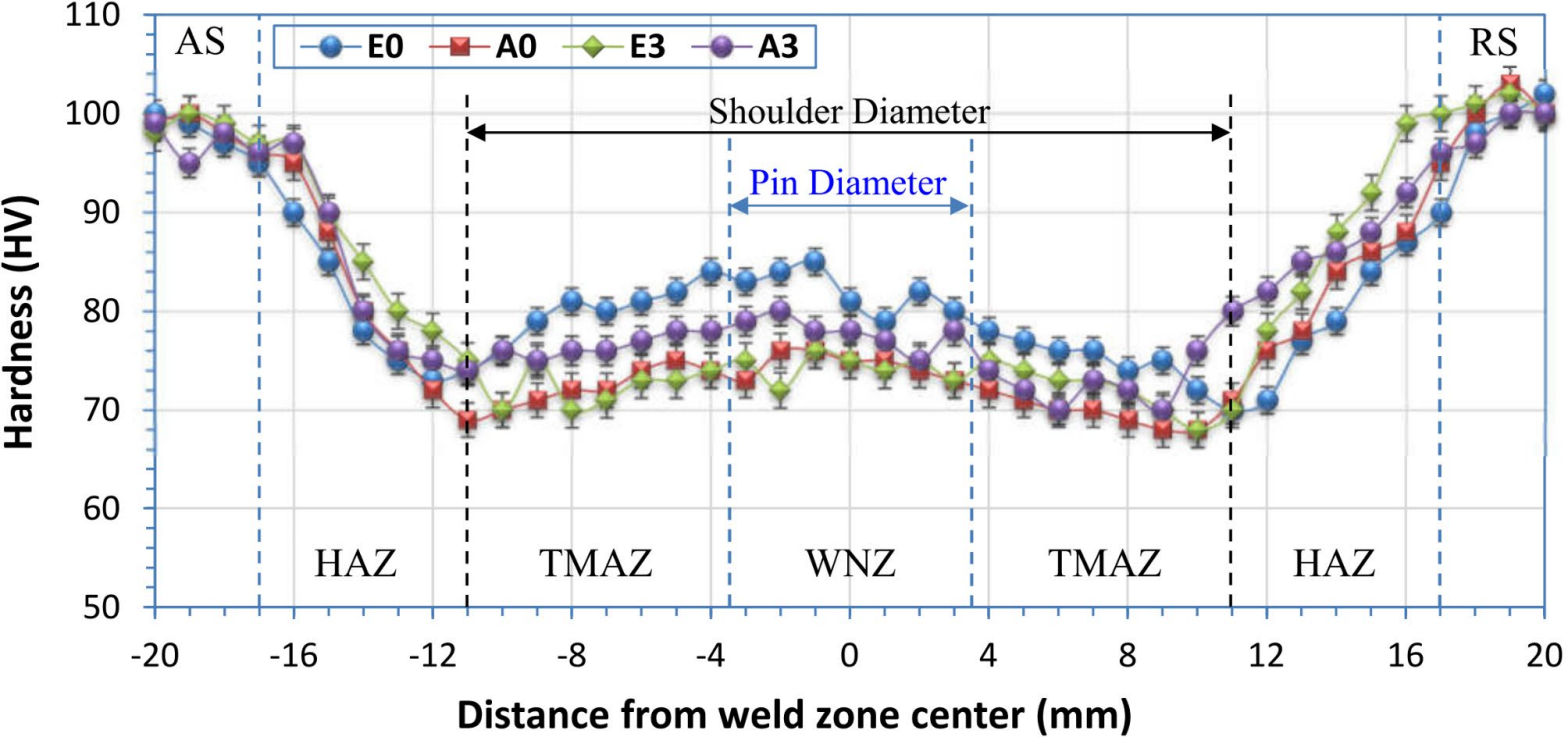
Hardness profile across cross sections of FSW joints.

Figure 12 illustrates the Vickers hardness (HV) survey on the cross-section of FSWed joints coded with E0, A0, E3, and A3 according to Table X. For all welded joints, typically, “W” shape profiles of hardness were observed for the FSWed joints, as shown in Fig. 12. It can be mentioned that the WZ in the FSWed joints revealed a hardness higher than the other regions of the TMAZ and HAZ. Furthermore, the hardness of the welded joints gradually decreased in the TMAZ and HAZ (Fig. 9). The WZ is characterized by a fine-grained microstructure^65–67^. In contrast, the HAZ and TMAZ exhibit varying degrees of thermal exposure and mechanical deformation, resulting in softer and larger grain sizes compared to grains than those in the WZ microstructure^68,69^. This difference in the microstructure of the FSW regions and hardness between the WZ, HAZ, and TMAZ is an essential aspect of the variation in the tensile properties of the FSWed joints. Among the various FSW regions, the TMAZ of the FSWed joints obtained a lower hardness. In the TMAZ, the temperature generated during the FSW diffused. In this region, the temperature led to incomplete recrystallization in the generated microstructure, leading to a reduction in hardness compared to the other regions. These hardness results were confirmed by previous studies conducted on heat-treatable aluminum alloys^60,70–73^. Scialpi et al.^73^ studied the effect of shoulder geometries on the hardness properties of AA6082-T6. They reported that the FSW process softened the material in the WZ, causing a reduction in hardness of approximately 35% in the weld zone, and the hardness in the cross-section of the welded joints obtained a “W” shape. Furthermore, the hardness in the WZ was higher than that in the TMAZ and HAZ. The effect of the FSW process on the properties of similar and dissimilar heat-treatable (AA6061-T6 and AA6082-T6) aluminum alloys was investigated by Moreira et al.^60^. They also concluded that the hardness profiles of similar joints (AA6061-T6 and AA6082-T6) and dissimilar joints (AA6061-T6/AA6082-T6) revealed a “W” shape with higher hardness in the WZ than in other regions of the TMAZ and HAZ. However, compared to the hardness of the AA6082-T6 initial plate, the hardness of the WZ revealed that the hardness of the WZ was remarkably lower than that of the AA6082-T6 initial plate, as shown in Fig. 12. Scialpi et al.^73^ indicated that the reduction in the hardness values of the WZ compared to the parent material is mainly due to the different differences between the microstructure developed during the FSW process and the nature of the microstructure of the initial plate. Moreira et al.^60^ reported that the precipitate of B”-Mg_5_Si_6_ was mainly responsible for the hardening of AA6082 aluminum alloys. During the FSW, the B”-Mg5Si6 phase dissolved in the WZ, TMAZ, and HAZ. Furthermore, fine precipitates of B”-Mg5Si6 were not detected in the WZ. In the current study, among all the welded joints, the highest WZ hardness of 82 HV was obtained for the E0 joints welded using the eccentric shoulder without tilting angles. This increase in the WZ hardness of E0 was confirmed by the grain-size analysis results, which confirmed the increase in the WZ hardness of E0 (Figs. 8 and 9**)**.

## Conclusions

This study examined the influence of an eccentric shoulder on the microstructural and mechanical properties of FSWed AA6082-T6. Two tools were employed, one with an eccentric shoulder and the other with an aligned shoulder, with the welding parameters kept constant. Empirical and theoretical analyses led to the following conclusions:

- The eccentric shoulder tool with a 0° tilt angle produced the finest grain structure in the weld nugget zone, with an average grain size of 1.63 μm, representing a 69% reduction compared with the base material. AA6082-T6 initial plate.
- The joints produced using the eccentric shoulder tool at a 0° tilt angle exhibited superior mechanical properties, achieving 89.7% of the ultimate tensile strength of the initial plate (216.5 MPa) and 68.8% of its elongation. The yield strength for this configuration was also notably high at 68.3 MPa, suggesting that the eccentric shoulder design effectively maintains joint integrity without requiring a tilt angle.
- The eccentric shoulder tool generated higher peak temperatures and heat inputs than the aligned shoulder tool, even without a tilt angle. This increased heat generation is attributed to the larger frictional area and dynamic orbit created by the eccentric design, which enhances material flow and mixing patterns.
- The results of this study suggest that the use of an eccentric shoulder tool in friction stir welding can simplify the process by eliminating the need for tool tilting, making it an attractive solution for industries such as aerospace, automotive, and shipbuilding. This approach has the potential to reduce the machine complexity and operational costs while maintaining or even improving the mechanical properties of welds.

## Data Availability

The datasets used and/or analyzed during the current study available from the corresponding author on reasonable request.

## Declarations

**Correspondence** and requests for materials should be addressed to A.R.S.E.

### Reprints and permissions information

is available at www.nature.com/reprints.
